# The impacts of computer adaptive testing from a variety of perspectives

**DOI:** 10.3352/jeehp.2017.14.12

**Published:** 2017-05-29

**Authors:** Tetsuo Kimura

**Affiliations:** Department of Clinical Psychology, Faculty of Social Welfare and Psychology, Niigata Seiryo University, Niigata, Japan; Hallym University, Korea

**Keywords:** Computer adaptive testing, Measurement efficiency, Learning self-efficacy, Motivation, Content balance

## Abstract

Computer adaptive testing (CAT) is a kind of tailored testing, in that it is a form of computer-based testing that is adaptive to each test-taker’s ability level. In this review, the impacts of CAT are discussed from different perspectives in order to illustrate crucial points to keep in mind during the development and implementation of CAT. Test developers and psychometricians often emphasize the efficiency and accuracy of CAT in comparison to traditional linear tests. However, many test-takers report feeling discouraged after taking CATs, and this feeling can reduce learning self-efficacy and motivation. A trade-off must be made between the psychological experiences of test-takers and measurement efficiency. From the perspective of educators and subject matter experts, nonstatistical specifications, such as content coverage, content balance, and form length are major concerns. Thus, accreditation bodies may be faced with a discrepancy between the perspectives of psychometricians and those of subject matter experts. In order to improve test-takers’ impressions of CAT, the author proposes increasing the target probability of answering correctly in the item selection algorithm even if doing so consequently decreases measurement efficiency. Two different methods, CAT with a shadow test approach and computerized multistage testing, have been developed in order to ensure the satisfaction of subject matter experts. In the shadow test approach, a full-length test is assembled that meets the constraints and provides maximum information at the current ability estimate, while computerized multistage testing gives subject matter experts an opportunity to review all test forms prior to administration.

## Introduction

Computer adaptive testing (CAT) is a kind of tailored testing, and is mostly based on item response theory (IRT). IRT is an extremely effective psychometric model that was formally proposed by Lord in 1980 [1]. Although CATs can still be constructed on the basis of classical test theory (CTT) [2-4], most CATs are constructed with IRT because it has several advantages over CTT in terms of test development, item analysis, and the scoring of test-takers. The most important advantage is that IRT places items and test-takers on the same scale.

CAT is a kind of computer-based testing that is adaptive to each test-taker’s ability level. An ideal CAT can provide each test-taker with a tailored test of a certain test length that may be different from others [5,6]. CAT has several major impacts on educational and psychological assessments, especially on proficiency and qualification tests.

The author will discuss the impacts of CAT from different perspectives in order to illustrate crucial points to keep in mind during the development and implementation of CAT. Additionally, the author will discuss a few more points that should be kept in mind during this process, such as question types, unidimensionality, and item exposure rate.

### Perspectives of test developers and psychometricians

Test developers and psychometricians often emphasize the efficiency and accuracy of CAT, comparing it to traditional linear tests based on CTT. CAT is time-saving, as it can shorten the test without losses in accuracy. If a test-taker answers the item correctly, the next item will be more difficult. In contrast, if the test-taker answers the item incorrectly, the next item will be easier. By selecting the appropriate levels of difficulty for items specific to each test-taker in this way, the information obtained from each individual item and the entire test itself is maximized.

CAT requires the following components: (1) a calibrated item bank, or a pool of questions calibrated with pretesting data; (2) a starting point, which is a rule to determine a specific starting point for the test-taker; (3) an item selection algorithm, which refers to rules to select the next item on the basis of the test-taker’s responses; (4) a scoring algorithm, which is a mechanism to estimate the test-taker’s ability and the measurement error; and (5) a termination criterion, which is a rule for terminating the test [7,8]. CAT begins with the assumption that the first 2 components, (1) and (2), are givens, then it repeats (3), (4), and (5) until the termination criterion component has been met. Although a considerable amount of research has been conducted over the last few decades on these particular aspects of CAT, the literature containing practical guidance on CAT development is scant [9].

Dr. Rudner’s on-line interactive mini-tutorial on CAT offers a simple CAT demonstration suitable for anyone who has not yet experienced CAT, but would like to [10]. This is the perfect place to become familiar with the inherent logic of CAT, to observe the behind-the-scenes calculations that are involved in the operation of CAT, and to obtain the opportunity to perform practical experiments with an actual CAT that selects an item adaptively from 64 multiple-choice questions on an eighth-grade mathematics test. The system provides the questions and the corresponding answers, so you can apply alternative choices and observe the results. The test starts after the user chooses his or her true ability in z-score units, in SAT units, or in percentiles. When testing, the system shows the informational applications of the 5 items that the computer thinks will optimize information about the test-taker’s ability level, the item response function for the corresponding item, and a standard error based on the current ability estimate. The test concludes when the “Done” button is pressed. The system then presents a history of the testing session, which includes the level of difficulty for each item, correct response probabilities, and whether the user gave the correct answer to a given item, while also providing standard errors and ability estimates. One can observe how the estimate of ability generally starts to mirror the preselected true ability score as the number of items increases, and how the standard errors for the estimate of the test-taker’s ability decreases as the number of items increases.

Empirical studies [6,7,11] have shown that CAT can reduce the number of items and testing time by 50% or more without deteriorating measurement precision, and that CAT is flexible enough to be configured to ensure that all test-takers are assessed with equal precision, regardless of the fact they may all be potentially presented with varying items.

### Perspectives of test-takers

Most CAT algorithms select items that each test-taker should have a 50% probability of answering correctly, since doing so maximizes test information and minimizes the number of items to be administered. However, many test-takers report feeling discouraged after taking such CATs. Some researchers have claimed that 50% is too low of a threshold for test-takers to retain their motivation, and that CAT should use easier items to imbue test-takers with a feeling of accomplishment [12,13]. According to a written survey conducted by the author, approximately 90% of test-takers found the test “difficult,” and approximately 60% also felt “discouraged” or “unsatisfied” with the experience. They claimed to usually feel “satisfied” when their scores exceeded 76/100, and “disappointed” when they were under 45/100. This suggested that the experience of taking a CAT could discourage test-takers and lead to counterproductive results, in terms of reductions in both self-efficacy and motivation for learning. Therefore, a trade-off exists between the psychological experiences of test-takers and measurement efficiency [14,15].

One solution to this problem is to manipulate the target probability of answering correctly in the item selection algorithm in CAT. To this end, the author developed a plug-in for Moodle, the world’s most popular open source learning management system, so that CAT can be administered on the system. This plug-in is called multidimensional (M)-unidimensional CAT (UCAT), and it was developed based on the program UCAT created by Linacre in 1987. M-UCAT has a function for controlling the target probability of answering correctly in the item selection algorithm, which the original UCAT did not have [16].

Obviously, if CAT were to select items that test-takers could answer correctly at a better than 50% probability, more items would have to be administered in order to maintain sufficient accuracy. The author used M-UCAT to administer different CATs, which selected items adaptively from the same item bank and differed with respect to target item difficulty and test length. (1) 16-item CAT with a 50% target probability of answering correctly, (2) a 25-item CAT with an 80% target probability of answering correctly, (3) a 19-item CAT with an 80% target probability of answering correctly for the first 8 items and 50% for the rest, (4) a 19-item CAT with an 80% target probability of answering correctly for the last 8 items and 50% for the rest, (5) and a 19-item CAT with an 80% target probability of answering correctly for both the first and the last 4 items and 50% for the rest. Theoretically, these 5 CATs obtained about the same amount of information from the test and ended with the same measurement precision (standard error, 0.5 logits). The ability estimation for the 5 groups was almost identical on average, with about the same standard deviation. At the end of all the CATs, the standard error of measurement reached as low as was theoretically expected on average, or even lower. Surprisingly, the actual percent correct for each CAT was lower than target probability of answering correctly. The decline in the standard error of measurement and the actual percent correct mainly occurred because the item bank had a left-skewed distribution of item difficulty [17,18].

Another disadvantage of CAT from the test-taker’s perspective is that a test-taker is not allowed to return to items already administered to change the answer, which is always possible in traditional standardized non-adaptive linear tests. The elimination of item review might increase the test-taker’s anxiety. If a test taker answers all the first few items incorrectly because of nervousness, the test is not self-correcting. Some researchers argue that CAT should start with relatively easy items [19].

### Perspectives of educators and subject matter experts

When educators and subject matter experts are introduced to the concept of CAT, they are often confused by the fact that each test-taker answers different questions in CAT, and that test-takers answer a much smaller number of items than on traditional linear tests. Their major concern is usually coverage and balance of subject matter content. Paper-based or compute-based linear tests, especially high-stake tests, such as university entrance examinations or qualification tests, are usually reviewed and revised elaborately by subject matter experts before the tests are administered. However, it is impossible to review the items presented on CATs in advance because CATs select items based on test-takers’ responses. Even after the administration of CATs, it is almost impossible to review items to check their content coverage and balance because there are so many different combinations. Accreditation bodies such as the Korea Health Personnel Licensing Examination Institute may be faced with a discrepancy between the perspectives of psychometricians and those of subject matter experts.

If a CAT selects items solely based on the test-takers’ ability, content balance and coverage may be easily distorted for some test-takers. There are also other nonstatistical specifications for CAT that we must consider, such as item format, answer keys, test length, and item enemies, which are pairs or clusters of items that are generally prohibited from appearing together on the same examination, since they could cue one another, or are too similar or redundant. In linear tests, any lapses of adherence to these parameters in the test forms are usually detected upon review by subject matter experts. However, in CAT this contingency is not present, and the item selection algorithm must be guaranteed to be able to automatically satisfy the specifications set. This is not an easy task.

The objective function of an ideal CAT is to optimize the statistical information in the test items at each test-taker’s current ability estimate. However, nonstatistical specifications, which we must take into account when developing a CAT, place constraints on the item selection procedure. Some researchers view this situation as an algorithm for constrained sequential optimization, where all nonstatistical specifications are constraints subject to the level at which the optimization has to take place [20].

Several different methods have been proposed to solve this problem over the last 2 decades: (1) item-pool partitioning [21], (2) the weighted-deviation method [22], (3) the maximum priority index method [22], (4) testlet-based adaptive testing [23-25], (5) multistage adaptive testing [26,27], and (6) adaptive testing with a shadow test [20,28]. The first 3 methods implement the necessary constraints through a modification of the item selection algorithm. The fourth and fifth methods implement the constraints directly into the test units in the pool from which the test is administered. The last method assembles a full-length test that meets the constraints and has maximum information at the current ability estimate, which is called a shadow test; however, only the item with the maximum information among the unused items in the shadow test is administered. After updating the ability estimate, the next shadow test is reassembled including the administered item(s).

If all nonstatistical specifications for the test can be explicitly coded and the item bank has a huge number of items with all possible combinations of values for the item parameters, test assembly using the shadow test approach always provides full-length tests with maximum information at each ability level. The more constraints need to be considered and controlled, the more items are necessary in the item bank. Subject matter experts are eager to review the entire test even if they know that the CAT system theoretically provides ideal full-length tests. The shadow test approach is psychometrically quite a sophisticated and flexible method, but it is not often chosen when changing from linear testing programs to adaptive programs with identical content specifications.

Multistage adaptive testing is the best approach for subject matter experts to review all completed testing material before administration in order to check the nonstatistical specifications, such as content coverage and balance, form length, item format, and item enemies. Many theoretical and empirical studies of computerized multistage testing have been conducted. Workshops and symposiums regarding computerized multistage testing are often held at major international conferences. “Computerized multistage testing: theory and applications” [29] is a comprehensive handbook in this field. Multistage adaptive testing has been widely adopted in the process of changing from linear testing to adaptive testing. The implementation of a multistage adaptive test in the Uniform Certified Public Accountant Exam in the United States is one of many good examples of this. The argument in its developmental stage is quite practical and thought-provoking for people who are considering implementing CAT in their assessments [30].

### Other issues

Aside from these issues, there are a few more things we should keep in mind when we develop and implement CATs: question types, unidimensionality, and the item exposure rate. CATs are basically not applicable to open-ended questions and items that cannot be calibrated easily. The IRT model that most CATs are based on assumes the unidimensionality of items, which means that all test items must measure a single trait. If it is desired to administer items that measure more than 1 trait, CAT should be developed based on multidimensional IRT [25,31-33].

The item exposure rate must be carefully controlled, because some items are chosen more frequently than other items, and some items may be memorized and passed on to other test-takers. Many procedures and approaches have been proposed so far. Some of these are: (1) the 5-4-3-2-1 randomization algorithm [34], (2) the Sympson and Hetter procedure [35], (3) the Davey and Parshall methodology [36], (4) the Stocking and Lewis unconditional multinomial procedure [37], and (5) the Stocking and Lewis conditional multinomial procedure [37]. Evaluation studies to compare these methods have been repeatedly conducted [38,39].

## Conclusion

This paper has reviewed the impacts of CAT from different perspectives to illustrate crucial points in the development and implementation of CAT. From the perspective of psychometricians, efficiency of measurement, which means shortening the test without losses in accuracy, represents the most salient impact of using CAT in an assessment. However, optimizing this parameter seems to have a negative impact on test-takers, in terms of reductions in both learning self-efficacy and learning motivation. In addition, most CAT systems deprive subject matter experts of the chance to review a full test either before or after it is administered. For subject matter experts, it is a major priority to check whether the nonstatistical specifications, such as content coverage and balance, form length, item format, and item enemies are satisfied in each test.

Regarding the negative impact on test-takers, the author proposes increasing the target probability of answering correctly in the item selection algorithm even if it consequently increases the number of items needed to maintain the same measurement precision. In order to satisfy subject matter experts’ desire to check the nonstatistical specifications in all testing material before administering a test, 2 different approaches have been introduced: CAT with a shadow test approach and computerized multistage testing. In the shadow test approach, a full-length test is assembled that meets all relevant constraints and has maximum information at the current ability estimate. Therefore, there is no need to review test forms by subject matter experts. However, it is not an easy task to code all nonstatistical specifications in advance. Computerized multistage testing gives subject matter experts an opportunity to review all test forms before they are administered. This is a major reason why multistage adaptive testing has been widely adopted while changing from linear testing to adaptive testing.

Moreover, question types, unidimensionality, and the item exposure rate were also briefly discussed, as they are also important issues in developing and implementing CATs. It should be noted that the scope of this paper is limited. For further information about CAT development and research, we suggest visiting the online bibliography created by the International Association of Computer Adaptive Testing (IACAT), which is actively maintained and updated with the latest research information and topics in CAT by a group of IACAT volunteers [40].

## Supplementary materials

Supplement 1. Audio recording of the abstract.
